# Spatiotemporal Patterns and Determinants of Grain Self-Sufficiency in China

**DOI:** 10.3390/foods10040747

**Published:** 2021-04-01

**Authors:** Yingnan Niu, Gaodi Xie, Yu Xiao, Jingya Liu, Yangyang Wang, Qi Luo, Huixia Zou, Shuang Gan, Keyu Qin, Mengdong Huang

**Affiliations:** 1 Institute of Geographic Sciences and Natural Resources Research, Chinese Academy of Sciences, A11 Datun Road, Chaoyang District, Beijing 100101, China; niuyn.19b@igsnrr.ac.cn (Y.N.); xiaoy@igsnrr.ac.cn (Y.X.); liujy.17b@igsnrr.ac.cn (J.L.); wangyy.18b@igsnrr.ac.cn (Y.W.); luoq.18b@igsnrr.ac.cn (Q.L.); zouhx.19b@igsnrr.ac.cn (H.Z.); gans.17s@igsnrr.ac.cn (S.G.); kyqin@qdio.ac.cn (K.Q.); huangmd.19s@igsnrr.ac.cn (M.H.); 2 College of Resources and Environment, University of the Chinese Academy of Sciences, 19 A Yuquan Road, No.19, Shijingshan District, Beijing 100049, China

**Keywords:** grain yield, grain consumption, grain self-sufficiency, spatiotemporal patterns, China

## Abstract

The pattern of grain self-sufficiency plays a fundamental role in maintaining food security. We analyzed the patterns and determinants of grain production and demand, as well as grain self-sufficiency, in China over a 30-year period. The results show that China’s total grain production, with an obvious northeast–southwest direction, increased by 63%, and yields of rice, wheat, corn, tubers, and beans increased by 16, 49, 224, 6, and 103%, respectively. The trends in ration and feed grain consumption changes at the provincial scale were roughly the same as at the national scale, with the ration consumption ratio decreasing and the ratio of feed grain consumption increasing. The ration consumption in Northwest China was relatively high, while the feed grain consumption rates in Shanghai, Guangdong, Beijing, Tianjin, and Chongqing were higher. Compared with ration and feed grain, the proportions of seed grain and grain loss were relatively small. China’s grain consumption mainly concentrated in the central and eastern regions of China. Total grain, rice, corn, wheat, tubers, and beans consumption in feed grain showed a northeast–southwest trend, with consumption centers all shifting southward in the 30-year period. Corn accounted for the largest proportion in feed grain, followed by beans. Urban feed grain and urban ration hot spot areas have gradually transferred from the northwest to southeast coastal areas. The hot spots of rural feed grain consumption and rural ration consumption remained almost unchanged, located in the south of the Yangtze River and Central and Southern China, respectively. The grain self-sufficiency level developed well in the study period, while the areas with grain deficit were Beijing, Tianjin, Shanghai, Zhejiang, Fujian, Guangdong, and Hainan. The areas with high supply and high demand were mainly located in Central and East China, the areas with high supply and low demand were mainly distributed in Northeast China, and the areas with low supply and low demand were mainly located in Western China. The pattern of self-sufficiency of corn in feed grain has remained basically unchanged; the areas with corn feed grain deficit were Central and Southeast China, while North China had corn feed grain surplus. Compared with corn feed, the surplus of soybean feed was relatively poor. Factor detector analysis revealed that in different periods, the same impact factor had different explanatory power in the supply and demand pattern, and the comprehensive consideration of any two factors will enhance the explanatory power of grain supply and demand pattern.

## 1. Introduction

Food security, which is a pivotal issue in the establishment of human civilization and development in the 21st century, is among the major challenges related to the sustainable development of human society, and has been concerned by more and more scholars [1,2,3]. As China has the world’s largest population, China’s food security has attracted extensive attention both at home and abroad [4,5,6], especially after Brown [7] put forward the question “Who Will Feed China?” The grain self-sufficiency, based on grain production and consumption, plays a fundamental role in maintaining food security. 

China feeds nearly 20% of the world’s population with less than 9% of the world’s arable land [8], contributing its share to world food security. China’s grain production capacity has steadily increased with the support of a series of policies and measures, including the red line of farmland protection, the establishment of well-facilitated farmland, the establishment of functional grain production zones and major agricultural product production reserves, and the construction of farmland water conservancy projects [9,10]. Between 1950 and 2017, grain production in China increased at a faster rate than population. Total grain production was 113 million tons in 1949, 305 in 1970, 462 in 2000, and 603 million tons in 2015 [11]. In addition to the changes in total grain yield, grain production in China has regional and temporal characteristics. With increasing urbanization, the main areas producing and marketing grain in China have changed significantly. From the perspective of regional geographical location, the status of grain production in some southeastern provinces has been gradually declining, while constantly strengthening in some northeastern and western provinces, which have gradually become the new main provinces and regions producing grain [12,13,14]. The traditional pattern of grain transportation from south to north in China was gradually replaced by the pattern of grain transportation from north to south, with the grain production center constantly moving from south to north and from east to center [15]. The shift in the grain gravity center to the north means that the northern region is experiencing more grain production pressure, which, to a large extent, will impact the original ecological balance and cause serious environmental problems, including the deterioration of cultivated land quality [16], e.g., elevated concentrations of trace elements [17,18], groundwater over-exploitation [19], cadmium contamination [20], environmental pollution, and other serious consequences [11,13]. These impacts will restrict the sustainability of China’s grain production increase [21].

Although increasing production can guarantee food security, it does not guarantee a sufficient availability of all the foods needed to satisfy consumer demand because of the different functions of food [22]. In China, for example, fast-paced socio-economic transformation has been accompanied by shifting diets toward higher shares of non-starchy foods [23], with per capita meat, milk, and egg consumption increasing 3.9, 10, and 6.9 times, respectively, between 1980 and 2010 [24]. The demand for animal products is projected to increase further in China [24,25]. As a result, livestock production will nearly double in the next few decades [24]. Such trends change dietary health risks but also contribute to a growing demand for feed grain. The two dominant components of commercial feed, maize, and soybean, are playing an increasingly important role in China’s feed supply chain [25]. The share of soybean in protein feed will increase from 70.6% in 2010 to 89.2% by 2030 [25]. Over 2000 to 2013, maize demand for feed in China increased by 69%, and will increased to 173 million tons in 2030 [25,26]. 

Whether China can create a balance between grain supply and demand appears to be the key component in its grain and food security strategy in the future [25]; thus, a comprehensive analysis of the patterns of grain self-sufficiency and its determinants in China is needed. Currently, most studies of grain supply and consumption in China have focused on food production patterns and their influencing factors [13,16], changes in food consumption patterns [24,25], demand for specific feed crops [27], and environment influences [13]. To the best of our knowledge, most studies evaluated the relationship between grain supply and demand from the perspective of total grain, but the patterns of various types of grain, e.g., ration, feed grain, seed grain, grain loss, and grain for industrial materials, were not identified. Discussions are lacking of the spatial and temporal evolution of the patterns of grain consumption from the two different perspectives of rural and urban areas, where food consumption patterns differ. In addition, there are few studies of the spatial heterogeneity and aggregation of grain self-sufficiency so it is impossible to study the regional differences in the same system, which is not conducive to the realization of the macro-control of grain supply and demand at the national level.

Hence, the three objectives of this study were, first, to identify the temporal and spatial evolution characteristics of grain yield in China; second, to explore the spatial-temporal patterns of grain consumption from the four aspects of the ration, feed grain, seed grain, and grain loss patterns in urban and rural areas; and third, to divide China into different grain supply and demand types. In the reminder of this paper, determinants of the pattern of grain self-sufficiency are described. This result deepens our comprehensive and systematic understanding of China’s grain production, consumption, and self-sufficiency patterns, and provides a reference for the formulation of China’s food security policy in the future.

## 2. Materials and Methods

The types of grain considered in this study include rice, wheat, corn, tubers, and beans. Considering the availability of data, food consumption includes ration, feed grain, seed grain, and grain loss. Due to the considerable differences in the types and quantities of grain consumption between rural and urban areas, ration and feed grain were divided into urban and rural consumption [28].

### 2.1. Data Sources

The grain types considered in this study include rice, wheat, corn, tubers, and beans. China’s grain output, grain sown area, urban and rural population, and per capita consumption of grain, meat, eggs, and milk in urban and rural areas in 1989, 1999, 2009, and 2019 were obtained from China Statistical Yearbook 1990, China Statistical Yearbook 2000, China Statistical Yearbook 2010, and China Statistical Yearbook 2020 [29,30,31,32], respectively. Seed sowing demand per unit area of rice, wheat, corn, and beans in 2009 and 2018 was obtained from Data Compilation of Cost and Benefit of Agricultural Products in China [33,34]. Due to the lack of urban and rural population data in 1989 and 1999, the urban and rural population data in these two years were replaced by the urban and rural population data in 1990 and 2000, respectively.

### 2.2. Ration Consumption

#### 2.2.1. Ration Consumption in Rural Areas

Ration consumption in rural areas is expressed as:

(1) $$G_{rit}=f_{\mathrm{rit}}\times P_{\mathrm{rit}}$$

where *G_rit_* is rural gross ration consumption in year *t* in area *i*; *f_rit_* and *P_rit_* are rural ration consumption per capita and the rural population of year *t* in area *i,* respectively. In this study, the values of *t* were 1989, 1999, 2009, and 2019. 

#### 2.2.2. Ration Consumption in Urban Areas 

Ration consumption in urban areas was calculated as follows:

(2) $$G_{\mathrm{uit}}=f_{\mathrm{uit}}\times P_{\mathrm{uit}}$$

where *G_uit_* is urban gross ration consumption in year *t* in area *i*; *f_uit_* and *P_uit_* are urban ration consumption per capita and the urban population of year *t* in area *i*, respectively. In this study, the values of *t* were 1989, 1999, 2009, and 2019. Due to the lack of *f_ui_*_1989_*, f_ui_*_1999_*,* and *f_ui_*_2009_*,* in this study, our solution was as follows:

(3) $$f_{\mathrm{uit}=}\frac{f_{ui2019}}{f_{Gu2019}}\times F_{\mathrm{Gut}}$$

where *f_uit_* is urban ration consumption per capita in area *i*, *f_ui_*_2019_ is urban ration consumption per capita of 2019 in area *i*, *f_Gu_*_2019_ is urban ration consumption per capita of 2019 in China, and *F_Gut_* is urban ration consumption per capita of *t* in China. The values of *t* were 1989, 1999, and 2009.

### 2.3. Feed Grain Consumption

#### 2.3.1. Feed Grain Consumption in Rural Areas

Feed grain consumption in rural areas was calculated as

(4) $$G_{\mathrm{frmji}t}=\sum_{j=1}^{7}\left(Q_{jt}\times \delta_{j}\times \gamma_{mj}\times P_{\mathrm{rit}}\right)$$

where *G_frmji_* is the total rural consumption amount of feed grain for type *m* in area *i* of year *t*; *m* is rice, wheat, corn, tubers, or beans; *Q_jt_* is the per capita consumption of product *j* in year *t*; $\delta_{j}$ is the ratio of forage and pork (the feed required for each kilogram of pork produced, 2.01), the ratio of forage and beef (the feed required for each kilogram of beef produced, 0.93), the ratio of forage and mutton (the feed required for each kilogram of mutton produced, 0.81), the ratio of forage and milk (the feed required for each kilogram of milk produced, 0.35), the ratio of forage and eggs (the feed required for each kilogram of egg produced, 1.72), the ratio of forage and poultry (the feed required for each kilogram of poultry produced, 1.62), and the ratio of forage and aquatic product (the feed required for each kilogram of aquatic product produced, 1.2), separately [28]; $\gamma_{mj}$ is the proportion of grain type *m* in forage *j* (Table 1) [28]; and *P_ri_* is the rural population in area *i*.

Due to the pork, beef, and mutton consumption in rural areas in 1989, 1999, 2009, and 2019 in the original data being combined, we calculated the pork, beef, and mutton consumption in 1989, 1999, 2009, and 2019 according to the proportions of pork, beef, and mutton consumption in urban areas in 2019, which were 90, 5.4, and 4.6%, respectively.

#### 2.3.2. Feed Grain Consumption in Urban Areas

Feed grain consumption in urban areas was calculated as

(5) $$G_{\mathrm{fumjit}}=\sum_{j=1}^{7}\left(Q_{jt}\times \delta_{j}\times \gamma_{mj}\times P_{\mathrm{rit}}\right)$$

where *G_fumji_* is the total urban consumption amount of feed grain for type *m* in area *i*; *m* is rice, wheat, corn, tubers, or beans; *Q_jt_* is the per capita consumption of product *j* in year *t*; $\delta_{j}$ and $\gamma_{mj}$ have the aforementioned meaning in Section 2.3.1; and *P_ri_* is the urban population in area *i*.

Since the data of meat, eggs, milk, and aquatic products consumption in various regions in 1989, 1999, and 2009 were missing, the same method as in Equation (3) was used to calculate the consumption of meat, eggs, milk, and aquatic products in each region. In addition, because the beef and mutton consumption in urban areas in 1989, 1999, 2009, and 2019 in the original data was provided as comprehensive statistics, we calculated the beef and mutton consumption in 1989, 1999, 2009, and 2019 according to the proportion of beef and mutton consumption in urban areas in 2019, which was 1.46.

### 2.4. Seed Grain Consumption

The amount of grain for seed was calculated by the sowing area of different grain types and seed demanding per unit area:

(6) $$G_{\mathrm{sjit}}=S_{\mathrm{jit}}\times A_{\mathrm{jit}}$$

where *G_sjit_* is the total seed consumption of grain *j* in area *i*; *S_jit_* is the seed consumption per unit area of grain *j*; *A_ijt_* is the planting area of grain *j* in area *i*; *j* represents rice, wheat, corn, or beans; and *t* represents 1989, 1999, 2009, or 2019. The amount of tuber seed is equal to 10% of tuber yield.

### 2.5. Grain Loss

Grain loss is determined by the grain yield and grain loss rate:

(7) $$G_{\mathrm{ljit}}=G_{\mathrm{ojit}}\times \delta$$

where *G_ljit_* is the grain loss of type *j* in area *i* of year *t*; *G_ojit_* is the yield of grain type *j* in area *i* of year *t*;* t* is 1989, 1999, 2009, or 2019; and *δ* is grain loss rate. In this study, we used a grain loss rate of 0.05.

### 2.6. Total Grain Consumption

The total grain consumption in area *i* of year *t* is defined as

(8) $$G_{\mathrm{total}\_ti}=G_{\mathrm{rit}}+G_{\mathrm{uit}}+G_{\mathrm{frmjit}}+G_{\mathrm{fumjit}}+G_{\mathrm{sjit}}+G_{\mathrm{ljit}}$$

where *G_total_ti_* is the total grain consumption in area *i* in year *t*; *G_rit_*, *G_uit_*, *G_frmjit_*, *G_fumjit_*, *G_sjit_*, and *G_ljit_* represent ration consumption in rural areas, ration consumption in urban areas, feed grain consumption in rural areas, feed grain consumption in urban areas, seed grain consumption, and grain loss in area *i* in year *t*, respectively. 

### 2.7. Total Grain Yield

The total grain yield in area *i* of year *t* is defined as

(9) $$G_{y\mathrm{ield}\_ti}=G_{\mathrm{rice}\_ti}+G_{\mathrm{wheat}\_ti}+G_{\mathrm{corn}\_ti}+G_{\mathrm{tubers}\_ti}+G_{\mathrm{beans}\_ti}$$

where *G_yield_ti_* is the total grain yield in area *i* in year *t*; *G_rice_ti_*, *G_wheat_ti_*, *G_corn_ti_*, *G_tubers_ti_*, and *G_beans_ti_* are the total yield of rice, wheat, corn, tubers, and beans in area *i* in year *t*, respectively.

### 2.8. Temporal and Spatial Pattern of Grain Yield and Consumption

#### 2.8.1. Analysis of the Direction of Ration and Feed Grain Yield and Consumption

Standard deviation ellipse (SDE) was first proposed to reveal the spatial distribution characteristics of geographical elements. It has been widely used in sociology, epidemiology, ecology, and other fields [35,36,37,38]. The SDE method quantitatively describes the centrality, distribution, directionality, and spatial morphology of geographical elements through spatial distribution ellipse, with the center, major axis, minor axis, and azimuth as its basic parameters [37]. The major half-axis of the ellipse represents the direction of data distribution, and the minor half-axis represents the range of data distribution. The larger the difference between the major half-axis and the minor half-axis, the more obvious the directionality of data. Conversely, the closer the major and minor half-axes, the less obvious the directivity. If the major and minor axes are equal, there is no directional feature. The shorter the minor half-axis, the more obvious the centripetal force. The longer the minor half-axis, the more discrete the data. Similarly, if the minor half-axis and the major half-axis are completely equal, the data do not have any distribution characteristics. The center point represents the center position of the whole data. 

#### 2.8.2. Hot-Spot Analysis in Ration and Feed Grain Consumption

Getis-Ord $G_{i}^{*}$ was used to explore the cluster patterns of ration and feed grain consumption in China. The Z-value represents where the data are strongly or weakly spatially clustered [39,40,41]. Getis-Ord $G_{i}^{*}$ is calculated as:

(10) $$G_{i}^{*}=\frac{\sum_{j=1}^{n}W_{ij}x_{j}}{\sum_{j=1}^{n}x_{j}}$$

For the convenience of explanation and comparison, a standardized value is calculated as follows:

(11) $$Z\left(G_{i}^{*}\right)=\frac{\sum_{j=1}^{n}W_{ij}x_{j}-\bar{X}\sum_{j=1}^{n}W_{ij}}{\sqrt{\frac{s^{2}}{n-1}\left(n\sum_{j=1}^{n}W_{ij}^{2}-{\left(\sum_{j=1}^{n}W_{ij}\right)}^{2}\right)}}$$

where $x_{j}$ is the ration and feed consumption of area *j*, *W_ij_* is the spatial weight, $\bar{X}$ is the mean value of $x_{j}$, *n* is the total number of areas, and *s*^2^ is variance. A significantly positive $Z\left(G_{i}^{*}\right)$ indicates that ration and feed grain consumption near area *i* is greater than the mean value (hot spot). In contrast, a significantly negative $Z\left(G_{i}^{*}\right)$ means ration and feed grain consumption around area *i* is lower than the mean value (cold spot).

### 2.9. Temporal and Spatial Pattern of Grain Supply and Demand

#### 2.9.1. Grain Surplus and Deficit

The grain surplus and deficit is defined as:

(12) GSD_ti=Gyield_tiGtotal_ti

where *G_SD_ti_* is the ratio between total grain production and total grain consumption in area *i* in year *t*. If *G_SD_ti_* is greater than 1, the region has a grain surplus; if *G_SD_ti_* is less than 1, grain is in short supply in this region. If *G_SD_ti_* equals 1, the grain supply and demand are in balance. 

#### 2.9.2. Division of Grain Supply and Consumption

The total grain output and total consumption of each region were standardized by the Z-score. The standardized total grain output and total grain consumption were used to represent the grain output on the x-axis and grain consumption on the y-axis, respectively. The four quadrants, A, B, C, and D, were divided, representing four regions: high supply–high demand, low supply–high demand, low supply–low demand, and high supply–low demand, respectively. 

### 2.10. Determinants of Grain Supply and Demand Pattern

Geodetector software (http://www.geodetector.cn/, accessed on 25 December 2020) was used to analyze the determinants of the grain supply and demand pattern. This geographic detector was first used in the field of health risk to assess the environmental risks to human health based on spatial variation analysis of the geographical strata of variables [42,43]. The geographical detector includes four detectors: Factor detection, interaction detection, risk detection, and ecological detection. Wang offered a detailed explanation of the principle behind the geographical detector [42,43]. In this study, we used factor detection and interaction detection to reveal the factors controlling the grain supply and demand patterns in China from 1989 to 2019. 

According to our literature research and the available data, we selected eight factors (Table 2) from the perspectives of grain production [44] and grain consumption [45]. Firstly, the factors influencing dietary differences between northern and southern regions were divided into two categories, which were 0 and 1. Secondly, the other factors were divided into five categories based on the natural discontinuity method.

Factor detection, based on *q*-value measurement, was used to detect the spatial differentiation of attribute Y (the ratio of grain supply to demand) and the extent to which the spatial differentiation of attribute Y is dominated by factor X. *q* is calculated as:

(13) $$q=1-\frac{\sum_{h=1}^{L}N_{h}\sigma_{h}^{2}}{N\sigma^{2}}$$

where *h* = 1, …, *L* is the strata of variables or factors; *N* denotes the number of provinces in the study area; *N_h_* is the number of provinces in strata *h*; $\sigma_{h}^{2}$ and $\sigma^{2}$ are the variances of the ratio of grain supply and demand in strata *h* and the study area, respectively. The value of *q* ranges from 0 to 1. The larger the *q* value, the more obvious the spatial differentiation of Y. If the stratification is generated by variable X, the larger the *q* value, the stronger the explanatory power of variable X to attribute Y, and vice versa. In extreme cases, a *q* value of 1 indicates that factor X completely controls the spatial distribution of Y, and a *q* value of 0 indicates that factor X has no relationship with Y. 

Interaction detection was performed to identify whether two determinants, when considered together, weaken or enhance each other or are independent in the development of the grain supply and demand pattern. A detailed explanation is given by Wang et al. (2010).

## 3. Results

### 3.1. Spatiotemporal Patterns of Grain Production in China

#### 3.1.1. Temporal and Spatial Evolution of Total Grain Yield

The grain yield at the national level showed that varieties of grain were trending upward (Table 3). China’s total grain production rose from 408 Mt in 1989 to 664 Mt in 2019, an increase of about 63%. Compared with yield in 1989, the yields of rice, wheat, corn, tubers, and beans in 2019 increased by 16, 49, 224, 6, and 103%, respectively.

**Figure 1:**
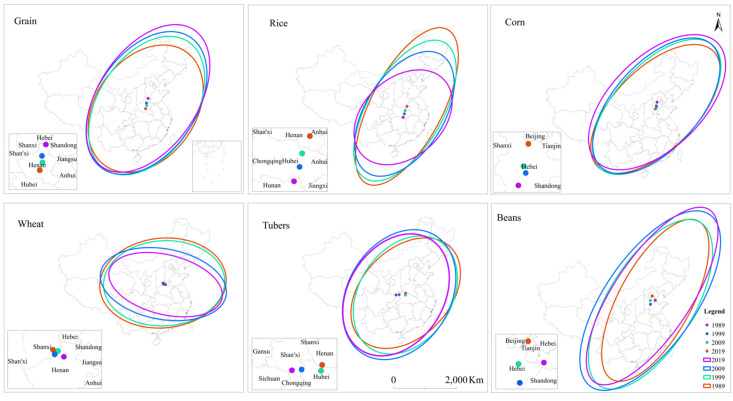
The spatial and temporal evolution of total grain yield and grain yield by variety in China from 1989 to 2019.

The directional evolution characteristics of grain production are shown in Figure 1. Total grain yield in China presented a pattern of northeast to southwest, and this direction gradually increased from 1989 to 2019 (Table 4). In the 30-year period, the center of China’s total grain yield was always located in Henan Province, but the central position has been moving northward. 

Similar to the pattern of total grain yield, the rice yield in China presented a pattern of northeast to southwest as well, and this directionality has increased over time. The center of rice output gradually shifted from the south of Henan Province to the south of Hubei Province. 

The spatial pattern of maize yield in China is northeast to southwest, but this direction has gradually weakened since 1999. The center of corn production is located in Hebei Province and has been gradually moving southward since 1989. 

A gradual strengthening pattern of southeast to northwest was found in the wheat yield since 1989. In 1989, the wheat yield center was located in the south of Shanxi Province. In 1999, it shifted to the southeast of Shanxi, and in 2009, it shifted to the southwest of Shanxi. Compared with 2009, the wheat yield center in 2019 shifted from the southeast to the north of Henan Province. 

The yield of tubers showed northeast–southwest in space, and the directionality gradually weakened from 1989 to 2009 and increased from 2009 to 2019. The yield center of tubers showed a trend of shifting to the south first and then to the west. The tuber yield center was located in the south of Henan in 1989, northwest of Hubei in 1999 and 2009, and the south of Shaanxi in 2019.

Compared with other crops, the northeast and southwest direction of beans yield is more obvious. From 1989 to 1999, the directionality weakened; from 1999 to 2019, it gradually increased. The beans yield center shifted south from 1989 to 2009, and then northwest from 2009 to 2019.

#### 3.1.2. Temporal and Spatial Evolution Pattern of Regional Grain Yield 

**Figure 2:**
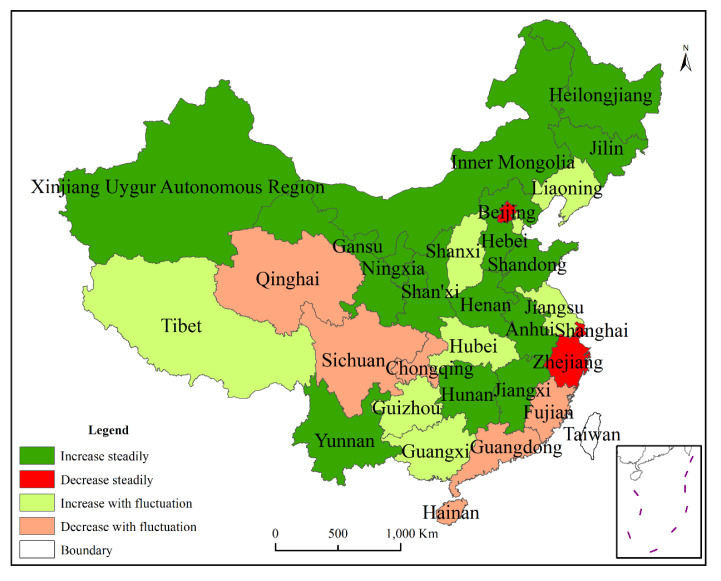
Division of total grain yield in China.

We further divided the changes in grain yield in the four periods into four types: increase steadily, decline steadily, increase with fluctuation, and decline with fluctuation (Figure 2). We observed that the steady increase in total grain yield was mainly distributed in Northern China. Steady decrease in total grain yield occurred in Beijing, Shanghai, and Zhejiang. Qinghai, Sichuan, Chongqing, Fujian, Guangdong, and Hainan experienced fluctuating grain yield decline. Tibet, Guizhou, Guangxi, Hubei, Jiangsu, Shanxi, Tianjin, and Liaoning showed an increasing and fluctuating trend in the total grain yield. 

### 3.2. Spatiotemporal Patterns of Grain Consumption 

#### 3.2.1. Temporal and Spatial Characteristics of Grain Consumption Structure

Table 5 shows that the trends in the consumption of ration and feed grain in China are opposing. The proportion of ration consumed dropped by 25%, from 80% in 1989 to 55% in 2019. However, the proportion of feed grain consumption rose considerably from 11% in 1989 to 31% in 2019, but there was little change in the amounts of seed grain consumption and grain loss.

**Figure 3:**
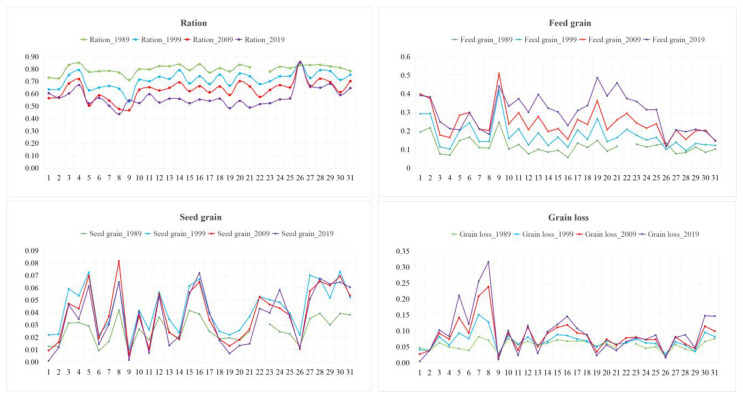
Proportion of grain consumption structures in different areas; 1, Beijing; 2, Tianjin; 3, Hebei; 4, Shanxi; 5, Inner Mongolia; 6, Liaoning; 7, Jilin; 8, Heilongjiang; 9, Shanghai; 10, Jiangsu; 11, Zhejiang; 12, Anhui; 13, Fujian; 14, Jiangxi; 15, Shandong; 16, Henan; 17, Hubei; 18, Hunan; 19, Guangdong; 20, Guangxi; 21, Hainan; 22, Chongqing; 23, Sichaun; 24, Guizhou; 25, Yunan; 26, Tibet; 27, Shan’xi; 28, Gansu; 29, Qinghai; 30, Ningxia; 31, Xinjiang.

The grain consumption at the provincial level (Figure 3) showed that the trend of ration and feed grain consumption change at the provincial level was roughly the same as at the national level, with the ration consumption ratio decreasing and the ratio of feed grain consumption increasing. The average ration consumption ratio in 1989, 1999, 2009, and 2019 was 80, 71, 63, and 57%, respectively. Tibet, Shanxi, Gansu, Qinghai, and Xinjiang had a relatively large proportion of ration consumption, while the ration consumption proportions in Chongqing, Inner Mongolia, Shanghai, Jilin, and Heilongjiang were low. Except for Tibet, the proportion of ration consumption in other regions decreased, with Heilongjiang (77 to 44%), Hainan (81 to 49%), Anhui (82 to 53%), Guangxi (83 to 54%), and Guangdong (78 to 48%) decreasing most. 

The average proportion of feed grain consumption in 1989, 1999, 2009, and 2019 was 12, 17, 24, and 30%, respectively. Shanghai, Guangdong, Beijing, Tianjin, and Chongqing had higher rates of feed grain consumption than in other areas in China. Compared with the regions with relatively large proportions of feed grain consumption, the regions with relatively low feed grain consumption were Tibet, Xinjiang, Gansu, Henan, and Shanxi. The ratio of feed grain consumption in Guangdong, Hainan, Zhejiang, Fujian, and Guangxi increased by more than 24%. 

Compared with ration and feed grain, the proportion of seed grain was relatively low, accounting for less than 10% of total grain consumption. Except for Beijing, Tianjin, Shanghai, Zhejiang, Fujian, Hunan, Guangdong, Guangxi, Hainan, Chongqing, and Tibet, the proportions of seed grain consumption in other regions showed upward trends, in which Inner Mongolia, Henan, Guizhou, Gansu, and Qinghai increased more. 

The average proportion of grain loss in 1989, 1999, 2009, and 2019 was 6, 7, 8, and 9%, respectively. In the 30-year period, grain loss occurred in larger proportions in Heilongjiang, Jilin, Inner Mongolia, Ningxia, and Henan. 

#### 3.2.2. Spatial and Temporal Characteristics of Total Grain Consumption and Grain Consumed in Feed Grain

**Figure 4:**
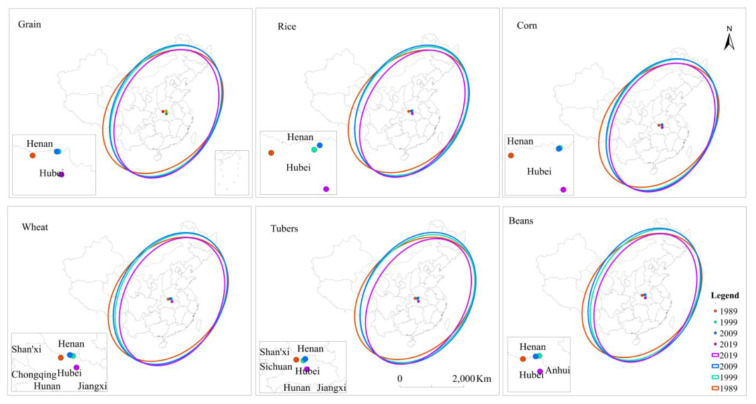
The spatial and temporal evolution of total grain consumption and grain consumption by varieties in feed grain in China from 1989 to 2019.

China’s grain consumption was mainly concentrated in the central and eastern regions of China. China’s total grain, rice, corn, wheat, tubers, and beans consumption in feed grain showed a northeast–southwest directionality in space, with consumption centers all shifting southward in the 30-year period (Figure 4). Table 6 shows that from 1989 to 2019, in addition to the weakening trend in the consumption direction of corn in feed grain, the consumption direction of other kinds of grain showed a strengthening trend.

In 1989, the center of total grain consumption was located in the south of Henan. It continued to move southward in Henan in 1999, and then moved westward from Henan to Hubei in 2009, and continued to move southward in Hubei in 2019. 

In 1989, the consumption center of rice in feed grain was located in the south of Henan. Since 1999, the center shifted southward to Hubei. Compared with the consumption center in 1999, the consumption center shifted to the northeast of Hubei in 2009, and continued to shift to the south in 2019.

The trends in consumption center changes of beans and corn in feed grain were consistent. From 1989 to 2009, the consumption centers of beans in feed grain were located in Henan province, but in 2009, the consumption centers moved westward, and in 2019, they moved southward to Hubei.

The trend of consumption center change of wheat in feed grain was almost the same as that of rice in feed grain. The only difference was that the consumption center of wheat in feed grain in 2009 moved northwest compared with that in 1999, while that of rice in feed grain was northeast. From 1989 to 2019, the consumption center of tubers in feed grain was located in Hubei, and the directional change trend was the same as that of the rice consumption center.

**Figure 5:**
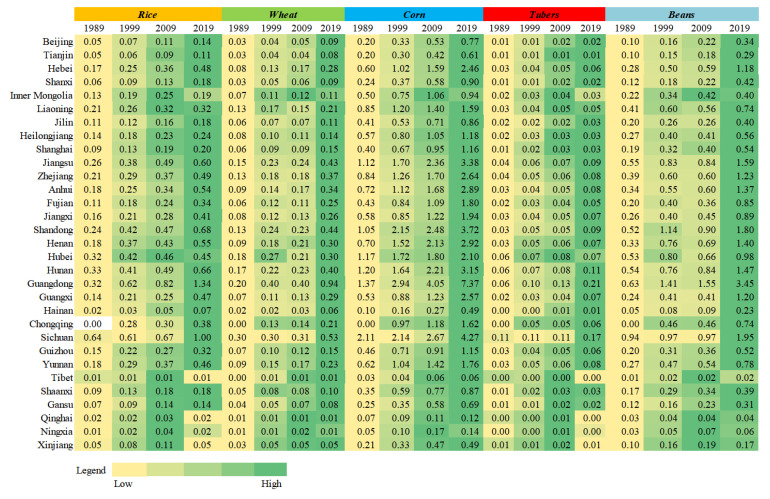
Rice, wheat, corn, tubers, and beans consumed in feed grain in China (unit: Mt).

Figure 5 that the consumption of various crops in feed grain showed an increasing trend, among which corn accounts for the largest proportion in feed grain, followed by beans.

#### 3.2.3. Spatial and Temporal Differences of Ration and Feed Grain Consumption between Urban and Rural Areas

**Figure 6:**
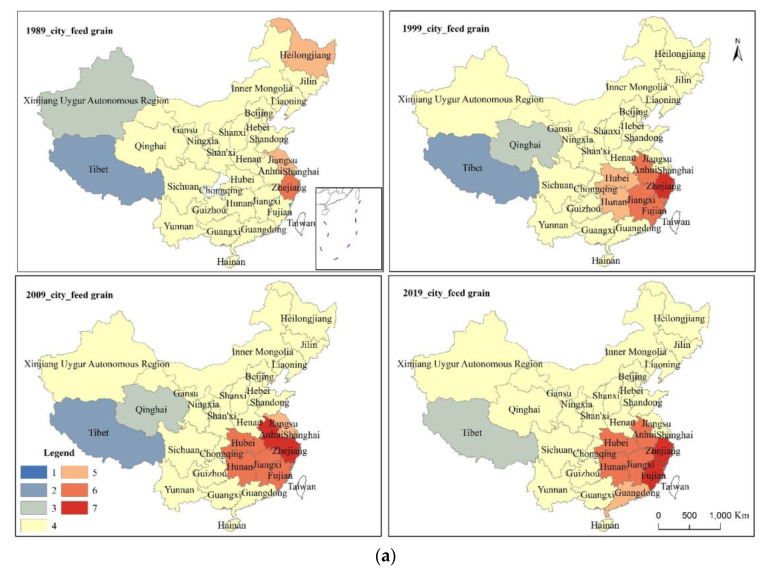

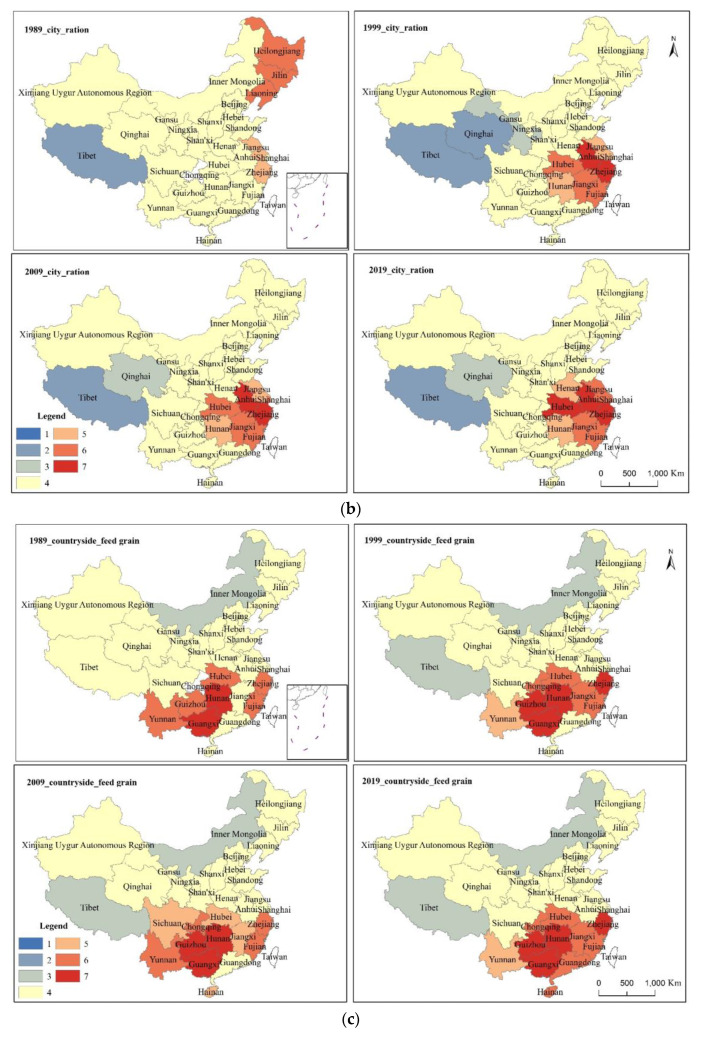

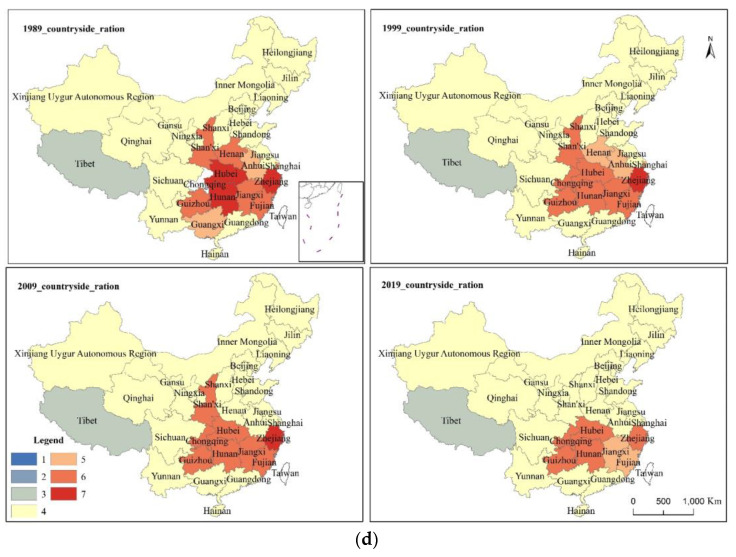
Cold and hot spots of ration and feed grain consumption in urban and rural areas in China (1, cold spot with 99% confidence; 2, cold spot with 95% confidence; 3, cold spot with 90% confidence; 4, not significant; 5, hot spot with 90% confidence; 6, hot spot with 95% confidence; 7, hot spot with 99% confidence).

The hot spots of urban feed grain consumption (UFC) in 1989 were Heilongjiang, Jiangsu, and Zhejiang. From 1999 to 2019, the UFC hot spots remained almost unchanged, mainly concentrated in Southeast China. In 1989, no first-class (99% confidence) hot spot of UFC was found. In 1999, Zhejiang was the first-class hot spot of UFC. In 2009, Anhui and Zhejiang provinces constituted the first-class hot spots of UFC. In 2019, the first-class hot spots of UFC were Fujian and Zhejiang. The cold spots were Tibet and Xinjiang, Tibet and Qinghai, Tibet and Qinghai, and Tibet, respectively. (Figure 6a)

In 1989, the hot spots of urban ration consumption (URC) were mainly distributed in Northeast China and Jiangsu, Shanghai, and Zhejiang. There was no significant change in the URC hot spots from 1999 to 2019. In 1999, the first-class (99% confidence) hot spot areas began to appear in Zhejiang and Anhui provinces. The first-class hot spots in 2009 were the same as in 1999; in 2019, the first-class hot spots were Hubei, Anhui, and Zhejiang. The cold spots of URC were Tibet, Tibet, Qinghai and Gansu, Tibet and Qinghai, and Tibet and Qinghai, respectively. (Figure 6b)

The hot spots of rural feed grain consumption (RFC) from 1989 to 2019 were mainly distributed in the south of the Yangtze River. From 1989 to 2009, the RFC hot spots expanded, successively including Chongqing, Jiangxi, Sichuan, and Hainan. In 2019, Guangdong became an RFC hot spot, but Sichuan withdrew. From 1989 to 2019, the first-level (99% confidence) hot spots of RFC were mainly concentrated in Guizhou, Hunan, Guangxi, and Zhejiang. The cold spot of RFC in 1989 was Inner Mongolia, whereas Tibet and Inner Mongolia together constituted the cold spots of RFC in 1999, 2009, and 2019. (Figure 6c)

From 1989 to 2009, the hot spots of rural ration consumption (RRC) remained almost unchanged, mainly distributed in Central and Southern China. In 1989, the first-level (99% confidence) RRC hot spots were Zhejiang, Hubei, and Hunan. Zhejiang was the first-level hot spot of RRC in 1999 and 2009. In 2019, no first-level hot spot was found. Tibet was the cold spot of RRC in 1989, 1999, 2009, and 2019. (Figure 6d)

### 3.3. Grain Supply and Demand Patterns

#### 3.3.1. Patterns of Total Grain Supply and Demand 

**Figure 7:**
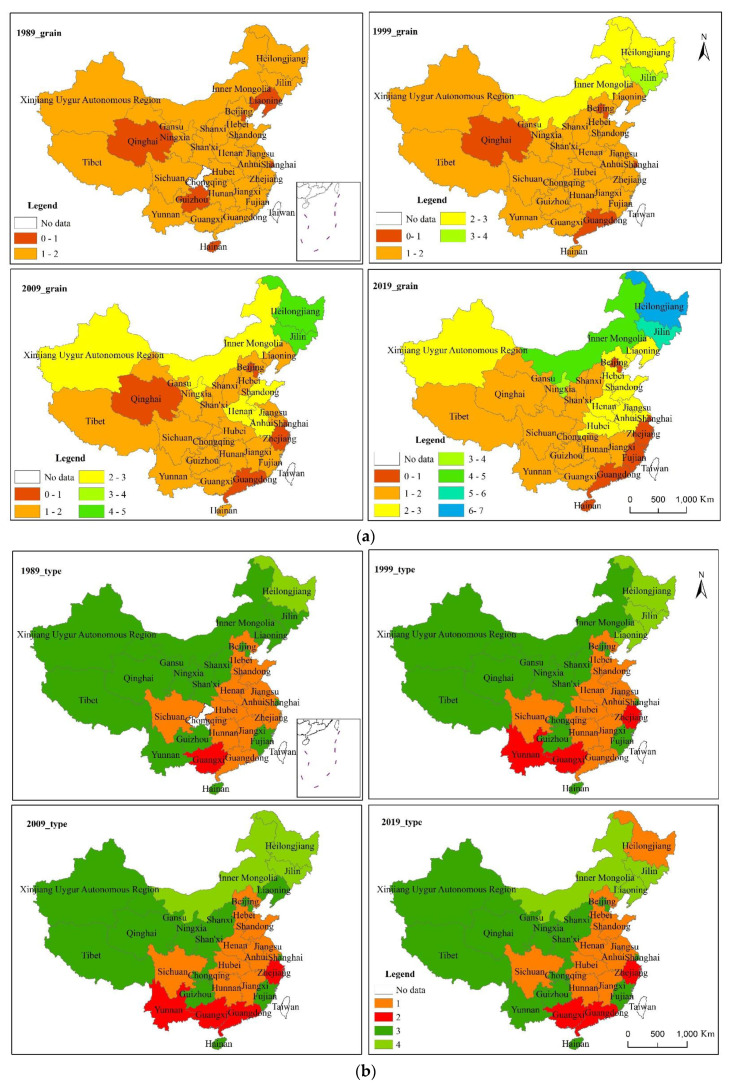
Grain supply and demand in China. (**a**) Proportion of grain supply to demand, the number indicates ratio of supply to demand. (**b**) Types of grain supply and demand. 1, high supply and high demand (H-H); 2, low supply and high demand (L-H); 3, low supply and low demand (L-L); 4, high supply and low demand (H-L).

On the whole, the grain self-sufficiency in the Northeast China Plain, North China Plain, Xinjiang, Inner Mongolia, and Ningxia developed well in the 30-year period. In 1989, the areas with grain deficit were Qinghai, Liaoning, Guizhou, Shanghai, Tianjin, and Hainan. In 1999, the areas with grain deficit were Qinghai, Beijing, Guangdong, Shanghai, and Tianjin. Qinghai, Beijing, Tianjin, Shanghai, Zhejiang, and Guangdong were grain deficit areas in 2009. In 2019, they were Beijing, Tianjin, Shanghai, Zhejiang, Fujian, Guangdong, and Hainan (Figure 7a). 

Figure 7b shows that the pattern of grain supply and demand in China did not change much in the 30 years. The areas with high supply and high demand (H-H) were mainly located in the middle and east of China, the areas with high supply and low demand (H-L) were mainly distributed in Northeast China, and the areas with low supply and low demand (L-L) were mainly located in Western China. Low supply and high demand (L-H) was initially located in Guangxi in 1989, and then Yunnan, Zhejiang, and Guangdong joined from 1999 to 2009. In 2019, Yunnan changed from a L-H to a L-L area.

#### 3.3.2. Self-Sufficiency Patterns of Feed Grain 

**Figure 8:**
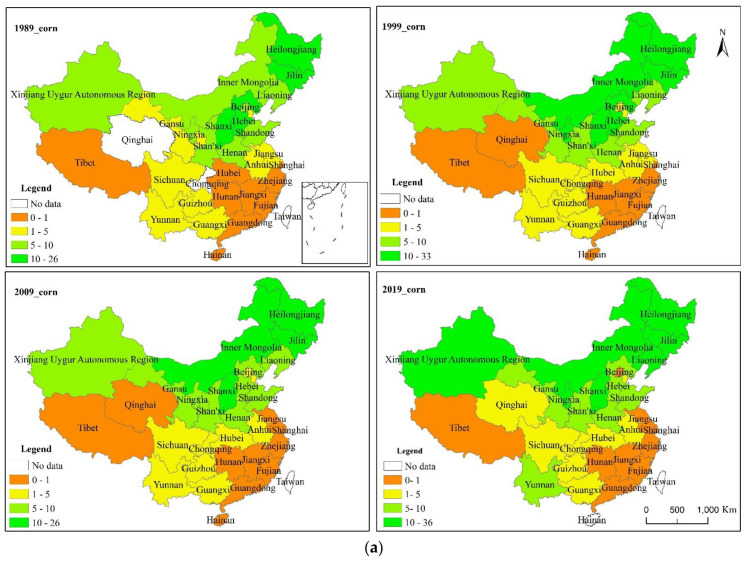

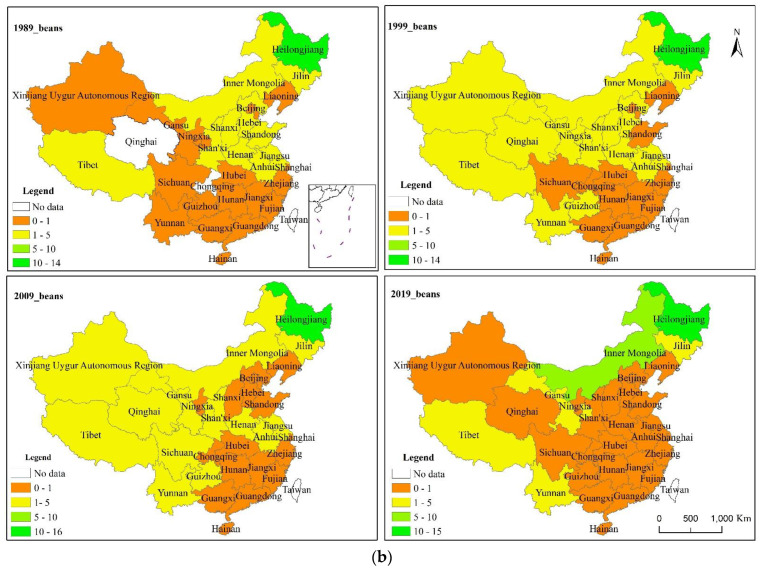
Relationship of corn and beans feed grain supply and demand in China, and the number indicates ratio of supply to demand.

In the 30 years, the pattern of self-sufficiency of corn in feed grain remained basically unchanged (Figure 8a): the areas with corn feed grain deficit were located in Central and Southeast China, while the regions with better corn feed grain surplus were located in North China. Compared with corn feed, the surplus of soybean feed was relatively low. From 1989 to 2009, China’s beans feed and grain shortage areas moved to the east of China, and from 2009 to 2019, bean feed shortage areas further expanded to the vast majority of China (Figure 8b).

### 3.4. Factors Controlling Patterns of Grain Self-Sufficiency 

The result of factor detector analysis (Table 7) revealed that in different periods, the explanatory power of the same impact factor on grain self-sufficiency pattern differed. In 1989, the most important factors controlling grain self-sufficiency pattern were effective irrigation area, proportion of urban population, and total power of agricultural machinery. In 1999, the prominent factors controlling grain self-sufficiency pattern were per capita consumption expenditure of urban residents, proportion of urban population, and per capita consumption expenditure of rural residents. In 2009, grain sown area, fertilizer consumption, and per capita consumption expenditure of rural residents were the top three factors dominating the grain self-sufficiency pattern. In 2019, grain sown area, total power of agricultural machinery, and per capita consumption expenditure of rural residents had strong explanatory power of the pattern of grain self-sufficiency.

**Figure 9:**
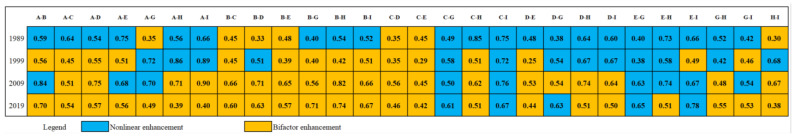
Interaction detection of factors controlling grain self-sufficiency pattern. A, proportion of urban population; B, grain sown area; C, effective irrigation area; D, fertilizer consumption; E, total power of agricultural machinery; G, dietary differences between the north and the south; H, per capita consumption expenditure of rural residents; I, per capita consumption expenditure of urban residents.

Interaction detection was applied to check whether two determinants of the patterns of grain self-sufficiency worked independently. The outcomes are presented in Figure 9. We considered the proportion of urban population and grain sown area in 1989 as an example to interpret the results. These two determinants accounted for 26 and 24% of the grain self-sufficiency pattern, respectively. However, the joint effect of the two factors was 59%. Thus, the proportion of urban population and grain sown area operating together enhanced the effects on the grain self-sufficiency pattern. Based on the analysis of Figure 8, we found that whenever any two factors operated together, a trend of enhanced interaction was observed.

## 4. Discussion

### 4.1. Grain Production in China

The evolution of grain production pattern is attributed to the joint action of natural factors, socio-economic factors, and technological progress [10,21,46]. The spatial distribution of cultivated land, grain sown area, and grain yield per unit area drove the formation of the spatial differences in grain yield in China. 

#### 4.1.1. Arable Land

**Figure 10:**
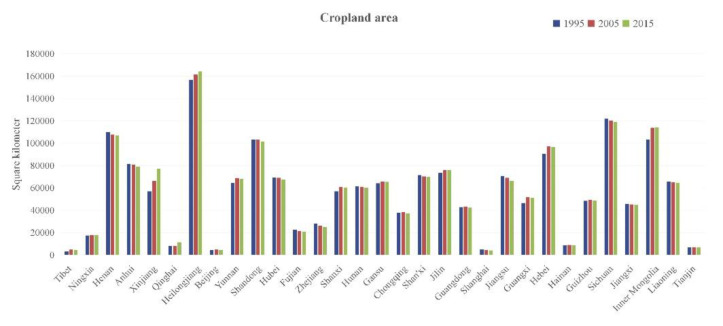
Changes in cultivated land area based on the results of land remote sensing monitoring in China (http://www.resdc.cn/, accessed on 3 January 2021).

Since the implementation of the policy of reform and opening-up in 1978, China’s industrialization has developed rapidly and township enterprises have been created, leading to the rapid increase in construction land and the continuous reduction in cultivated land [10,47], especially in China’s Yangtze River Delta, Pearl River Delta, and the Beijing–Tianjin–Hebei region (Figure 10). In addition to urbanization, China’s arable land is also facing serious challenges from soil erosion and desertification. China’s soil erosion area has reached 1.5 million square kilometers, desertification area is 176,000 square kilometers, and another 158,000 square kilometers are at risk of desertification; and if, according to UNEP’s criteria, a 25% drop in land productivity is the threshold for desertification, then 69% of China’s non-irrigated arable land is desertification, which brings stern challenges to grain production in China [48]. Moreover, at the end of the 20th century, Sichuan, Shaanxi, and Gansu provinces led the pilot project of returning farmland to forest, which enabled the return of farmland to forest in China [49]. However, to ensure the dynamic balance of cultivated land quantity, the Chinese government issued a series of policies implementing the system of compensation for cultivated land to ensure the quality of total cultivated land, such as the National Land Development and Consolidation Plan (2001–2010). Land consolidation includes agricultural land consolidation, high standard farmland construction, and basic farmland protection. Statistics showed that from 1997 to 2015, the country has arranged nearly 20,000 land consolidation projects, supplementing 22.76 million ha of arable land with an annual average of 252.9 million ha of arable land. Between 2001 and 2015, China added 2.77 million ha of arable land through land consolidation and built more than 13 million ha of high-quality basic farmland [47]. 

#### 4.1.2. Water Resources

Scarce water resources have been the main limiting factor of agricultural development in many areas of China. There are great differences in water resources between the south and the north of China. The area of cultivated land in the north accounts for 59.6% of the whole country, and the population accounts for 44.3%, while the water resources only accounts for 14.5%. Among them, the population and cultivated land account for 34.7 and 39.4% respectively, and the water resources only accounts for 7.6% in the Huang Huai Hai region; 84% of the water resources are in the southern region, where the population accounts for 53.6% and the cultivated land accounts for 34.7% [50]. In addition, the pollution level of Haihe River, northwest Yellow River, Liaohe River, and Huaihe River is far higher than the national average. However, the seriously polluted Huang Huai Hai area is responsible for 39.4% of China’s arable water [50].

#### 4.1.3. Income from Grain Planting

The income of agricultural production is the key factor affecting agricultural production in China. China’s urbanization process forces the rural youth and middle-aged labor force to transfer to the city and enter non-agricultural fields of employment, resulting in the reductions in time-consuming and laborious wheat and rice planting area, while the relatively time-efficient and labor-effective corn planting area increases [49]. In addition, affected by the rising prices of raw materials, fuels, production power, and labor and land costs, the cost of grain production has risen, which caused the decline of the average benefit of grain production per unit area [51,52]. From 2006 to 2018, the average total production cost of rice, wheat, and corn increased from 444.92 RMB (Legal currency of the people’s Republic of China) yuan per mu to 1093.65 RMB yuan per mu. During the same period, the average labor cost of the three main grain production increased from 151.96 RMB yuan per mu to 419.24 RMB yuan per mu, and the average land cost increased from 68.25 RMB yuan per mu to 224.86 RMB yuan per mu [51]. 

Regional grain support policies, e.g., grain market system reform plan, national and provincial grain risk funds, commodity grain production bases, the rice bag project, grain production subsidies, and implementation of agricultural tax reductions and exemptions, are being used to change the grain production pattern by fully mobilizing the farmers’ enthusiasm for grain production in the northeast and central regions. However, due to the low comparative benefits of grain production in the developed areas along the southeast coast, under the open grain market policy, the production enthusiasm has declined [10]; in some other areas, in order to increase their income, farmers choose to plant high-yield cash crops instead of food crops, and to plant single-season food crops, resulting in the continuous decline in the multiple cropping index [53], and thus shifting the focus of grain production in China

#### 4.1.4. Aging Population and Rural Population to Towns

China’s grain production is facing the severe challenge of population development. At present, China is in the period of ultra-low fertility, the end of population growth, the deepening of aging and more active population migration [54]. Due to the aging of rural population and the flow of rural young and middle-aged population to the city, most of the people engaged in agricultural production in rural areas are middle-aged and old people. However, the physical strength of old farmers is declining and they are not competent for heavy physical work. At the same time, the old farmers generally have low education level, conservative thinking and poor ability to accept new things, which is not conducive to modern agricultural technology and mode of production [55,56,57,58]. However, other studies show that the aging of rural population has no negative impact on China’s food production. In China, small-scale farmers usually plant the same crop and use the same technical measures in the field production. In fact, there is some form of collective decision-making, which indicates that the planting decision and crop production technology in the field agricultural production are highly imitative, which reduces the importance of human capital of workers [59,60]. Other studies have found that in the actual production of field crops, almost all the processes or key links with high labor intensity have been mechanized, and when the non-agricultural employment time of young and middle-aged male labor force increases, farmers will be more likely to rely on agricultural machinery “outsourcing” services rather than holding small agricultural machinery. The development of agricultural machinery service market improves the substitution degree of agricultural machinery for labor. Thus, it reduces the labor force requirements of agricultural production [59,61].

#### 4.1.5. Global Warming

Global warming and climate change have brought great challenges to agriculture [62]. In China, average temperature has increased in the last several decades since 1980s, which influencing crop phenology and yield across China [63,64,65]. The increase in heat resources caused by climate warming brings forward the spring phenology of crops and prolongs the growing period. Adequate heat during the growth period promoted stable and high yield of crops to some extent. However, the degree and trend of climate warming vary in different regions, and so do the temporal and spatial pattern of precipitation changes in different regions. Therefore, the increasing uncertainty of climate change will further increase the frequency and intensity of agricultural natural disasters, which will endanger the utilization of crop production potential [65]. Moreover, climate change has changed the spatial and temporal distribution pattern of heat in China, thus affecting the cropping system and structure of crops [65]. In addition, climate change will affect crop quality [65]. The increase in CO_2_ concentration will increase the absorption of carbon and reduce nitrogen, increase the ratio of carbon to nitrogen in crops, and decrease the protein content, which will reduce the quality of crops. Climate change leads to the occurrence, development and prevalence of crop diseases and insect pests. From 1961 to 2010, climate change led to the expansion of agricultural pests and diseases in China and the aggravation of their damage. The occurrence area of agricultural diseases and insect pests, diseases and pests in China increased by 5.38-fold, 7.27-fold, and 4.72-fold, respectively, from 1961 to 2010 [65].

#### 4.1.6. Agricultural Management and Investment

The development of agricultural mechanization; the construction of irrigation and water conservancy facilities; the use of agricultural plastic film, chemical fertilizers, and pesticides; and the breeding of varieties are important factors contributing to the improvement of China’s grain production [46,66,67,68,69]. Since the 1980s, groundwater has been developed by drilling in Northern China, and the effective irrigation area has been rapidly expanded. From 1990 to 2005, 2,049,000 wells were added in the whole country, 90% of which were concentrated in the northern region. Among them, Hebei, Shandong, and Henan provinces in the Huang Huai Hai Plain added 1,270,000 wells, accounting for 62% of the national increase, making this area the most concentrated continuous well-irrigation area in China [10]. The effective irrigation area in China increased by 44.03%, from 47,403,100 ha in 1990 to 68,271,600 ha in 2018 [70,71]. The development of irrigation in Northern China has improved the ability of agriculture to resist natural disasters while creating conditions for the popularization and application of advanced agricultural technologies, such as improved seed, fertilization, and cultivation [10]. Fertilizer use has more than doubled from 2.6 Mt in 1990 to 5.7 Mt in 2018, and pesticide use increased by 84.17%, from 1.09 Mt in 1995 to 2.00 Mt in 2018 [72,73]. In addition, the application and popularization of agricultural film have considerably increased the multiple cropping index in Northern China. Many one-year cropping areas in Northern China can produce two or even three crops per year [10]. The area covered by plastic film increased by 173.60%, from 6,493,000 ha in 1995 to 17,764,700 ha in 2018 [71,72]. Since the 1990s, three-dimensional agriculture in Northern China has developed rapidly. The three-dimensional planting of spring wheat intercropping corn, maize inter-cropping legume crops, and corn interplanting potato has become the main optimization form of the current single-cropping grain crop planting area in North China. This vertical planting mode can increase the annual grain yield by about 20% [10]. Thus, the productivity of land has been significantly increased and the grain yield has increased rapidly. However, while increasing grain production, a series of ecological and environmental problems have arisen, such as groundwater overdraft in North China and non-point source pollution caused by the use of chemical fertilizers and pesticides [73,74,75].

### 4.2. Grain Consumption Patterns in China

**Figure 11:**
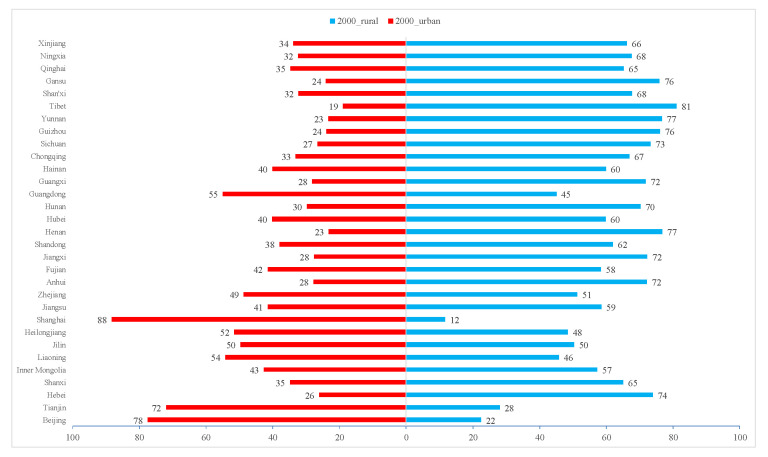

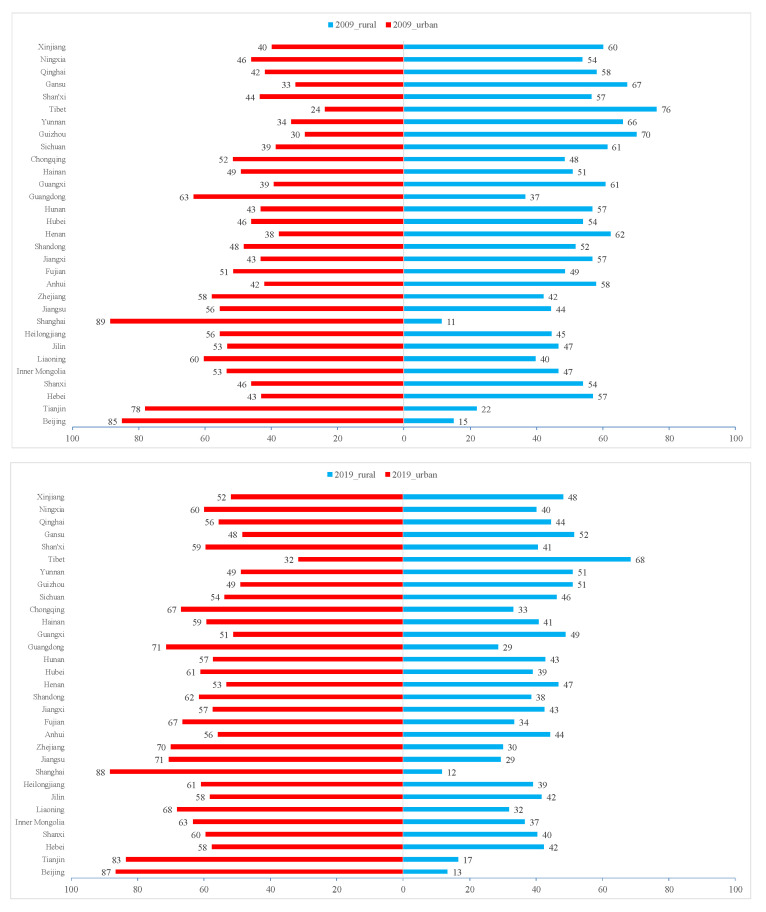
Proportion of urban and rural population in China in 2000, 2009, and 2019.

The change in food consumption demand is the direct factor driving the patterns of grain supply and demand balance. China’s urbanization process has accelerated since the 1990s, with the proportion of urban population increasing significantly (Figure 11), the income level of residents continuing to improve, and residents’ understanding of dietary nutrition knowledge deepening, leading to residents’ diet gradually changing to food safety and nutrition, which has promoted the diversified development of food consumption demand, resulting in the increase in feed food demand [76]. In the process of urbanization, workers have migrated from rural to urban areas, resulting in migrant workers’ original food consumption behavior transitioning to more closely mimic the consumption characteristics of urban residents, which will increase the demand for food by migrants in the city [53]. 

### 4.3. Policy Implications 

#### 4.3.1. Food Consumption Structure and Dietary Behavior Guidance

With the improvement in living standards of Chinese residents, the dietary structure of urban and rural residents has changed significantly [77], the intake of rations has decreased significantly, and the consumption of animal food, especially pork, has increased significantly. With the change in dietary structure, the nutritional status of residents also changed. The intake of heat energy and nutrients increased significantly, the proportion of fat intake increased year by year, and the proportion of carbohydrate intake decreased year by year. The change in dietary pattern has produced a series of effects. Chronic diseases related to nutrition, such as heart disease, cerebrovascular disease, and tumors, have become the three main causes of death. Therefore, in the future, China should strengthen nutrition education, correctly guide food consumption, correct residents’ bad food consumption habits, and establish a new concept of reasonable, balanced, and appropriate food consumption.

#### 4.3.2. Regional Coordinated Development of Grain Consumption

Significant differences exist in food consumption between urban and rural areas and between regions in China. China is facing the dual challenges of malnutrition and overnutrition. In the future, we should further promote regional food resource sharing, fully consider the characteristics and differences of different low pressure, and formulate scientific food development strategies.

#### 4.3.3. Optimization of the Layout of Grain Production

From a nationwide perspective, the center of grain production shows a northward trend, which means that the impact of national grain production on Northern China is increasing, with the pressure increasing on the resources and environment in Northern China. From the point of view of water resources, areas experiencing increase in grain production scale in the north are also experiencing prominent water resources problems in China. The shift of the center of grain production aggravates the pressure on the water supply in Northern China, diverting a large amount of water from the ecological environment, resulting in an increasing regional ecological deficit. Therefore, a reasonable pattern of grain production in line with the natural resources must be urgently established.

At present, China’s food supply is based on the production guiding consumption mode of production and consumption, rather than the ideal dietary nutrition mode of residents. The production of food structure cannot meet the needs of residents and improve dietary nutrition. Often, the products needed by residents are not available or are hard to buy on the market. In the future, we should further adjust and optimize China’s agricultural industrial structure and establish an agricultural industrial structure adjustment mode with nutrition and health as the main goal to coordinate residents’ consumption structure, nutritional diet structure, and food production structure, to promote the structural adjustment.

#### 4.3.4. Countermeasures of Adapting Agricultural Production to Climate Change in China

Climate change has greatly changed the spatial and temporal distribution characteristics of climate resources in China, which has put forward the requirements for China’s agricultural production, especially cropping system. In the face of climate change and changes in crop varieties, China’s agricultural production should seek advantages and avoid disadvantages, avoid risks of extreme weather and climate disasters, mitigate the adverse effects of climate change, effectively guarantee national food security, and achieve sustainable agricultural development.

### 4.4. Limitations and Directions for Future Work 

In this study, we quantitatively explored the spatial-temporal evolution characteristics of and factors influencing the grain supply and demand balance patterns in China. The results help to identify the problems with and limitations of grain production in different regions, which can be used to optimize the structure and spatial layout of grain production. However, this study has certain limitations. First, some key factors that influence grain supply and demand balance pattern were not included in the Geodetector analysis, such as agricultural disasters, number of people engaged in food production, etc. Second, this study of the pattern of grain supply and demand balance in China was based on the provincial scale, but resource endowment, agricultural investment, and residents’ income differ amongst the different regions of the same province; therefore, an analysis from the provincial scale cannot describe the micro differences in grain supply and demand pattern. Third, in the context of globalization and international market trade, China’s food security is related to international food trade. However, due to the uncertainties of distribution mechanism of China’s grain trade between domestic region is still unclear, we did not take into this aspect. So, in future research, we should pay attention to multi-scale correlations, take into food trade, and describe the factors influencing grain supply and demand as comprehensively as possible.

## 5. Conclusions

This finding indicated that China’s grain production, with obvious northeast–southwest direction, has increased in the studied 30-year period. Ration consumption ratio decreased, while the ratio of feed grain consumption increased. The ration consumption in Northwest China was relatively high, while the feed grain consumption rates in Shanghai, Guangdong, Beijing, Tianjin, and Chongqing were higher. Compared with ration and feed grain, the proportion of seed grain and grain loss were relatively small. Total grain, rice, corn, wheat, tubers, and beans consumption in feed grain showed a northeast–southwest directionality in space, with consumption centers all shifting southward in the 30 years. Corn accounted for the largest proportion in feed grain, followed by beans. Urban feed grain and urban ration hot spots gradually moved from northwest to southeast coastal areas. The hot spots of rural feed grain consumption and rural ration consumption remained almost unchanged, mainly located in the south of the Yangtze River and Central and Southern China, respectively. The grain self-sufficiency ratio developed well in the 30 years, while the areas experiencing grain deficit were Beijing, Tianjin, Shanghai, Zhejiang, Fujian, Guangdong, and Hainan. The areas with high supply and high demand were mainly located in the middle and east of China, the areas with high supply and low demand were mainly distributed in Northeast China, and the areas with low supply and low demand were mainly located in Western China. The pattern of self-sufficiency of corn in feed grain remained basically unchanged, among which the areas with corn feed grain deficit were located in Central and Southeast China, while North China had better corn feed grain surplus. Compared with corn feed, the surplus of soybean feed was relatively low. In different periods, the same impact factor had different explanatory power on the grain self-sufficiency pattern, and the comprehensive consideration of any two factors enhanced the explanatory power of the grain self-sufficiency.

## Figures and Tables

**Table 1:** Proportions of different types of grain in different forage (%).

	Rice	Wheat	Corn	Tubers	Bean Meal
Pig feed	15.77	6.7	43.57	15	15
Beef cattle feed	0	5.00	26.25	0	0
Sheep feed	0	5.00	26.25	0	0
Cow feed	0	0.7	31.40	0	0
Poultry feed for egg	6.20	2.7	40.40	0	20
Poultry feed for meat	2.32	2.33	50.82	0	20
Aquatic product feed	0	7.08	23.39	0	10

Note: The conversion of tubers to final grain tuber consumption needs to be multiplied by 0.2, and the conversion coefficient of bean meal to beans is 1.25.

**Table 2:** Selection of factors influencing grain supply and demand pattern.

	Factor	Variable
Grain yield	Resource endowment	Grain sown area
	Irrigation and water conservancy	Effective irrigation area
	Agricultural material input	Fertilizer consumption
	Agricultural mechanization level	Total power of agricultural machinery
Grain consumption	Urbanization level	Proportion of urban population
	Dietary differences	Dietary differences between the north and the south
	Living standard of residents	Per capita consumption expenditure of rural residents
		Per capita consumption expenditure of urban residents

**Table 3:** Gross grain yield in China (unit: Mt).

Year	Grain	Rice	Wheat	Corn	Tubers	Beans
1989	408	181	99	87	29	11
1999	508	199	127	139	38	20
2009	531	196	127	179	31	20
2019	664	210	148	281	30	22

**Table 4:** Oblateness of the standard deviation ellipse of grain yield.

	Grain	Rice	Corn	Wheat	Tubers	Beans
1989	0.285	0.289	0.500	0.292	0.315	0.599
1999	0.363	0.440	0.500	0.298	0.275	0.551
2009	0.367	0.524	0.480	0.448	0.259	0.564
2019	0.400	0.588	0.458	0.486	0.270	0.627

**Table 5:** Grain consumption structure in China (unit: Mt).

Year	Ration	Proportion	Feed Grain	Proportion	Seed Grain	Proportion	Grain Loss	Proportion
1989	254	80%	36	11%	9	3%	19	6%
1999	238	71%	53	17%	149	5%	25	7%
2009	181	63%	67	24%	12	4%	26	9%
2019	180	55%	103	31%	12	4%	33	10%

**Table 6:** Oblateness of the standard deviation ellipse of grain consumption.

	Grain	Rice	Corn	Wheat	Tubers	Beans
1989	0.293	0.291	0.293	0.287	0.284	0.297
1999	0.294	0.284	0.295	0.283	0.266	0.303
2009	0.277	0.275	0.280	0.272	0.264	0.276
2019	0.303	0.328	0.292	0.299	0.328	0.319

**Table 7:** Factors controlling grain self-sufficiency pattern based on Geodetector. A, proportion of urban population; B, grain sown area; C, effective irrigation area; D, fertilizer consumption; E, total power of agricultural machinery; G, dietary differences between the north and the south; H, per capita consumption expenditure of rural residents; I, per capita consumption expenditure of urban residents.

Year	A	B	C	D	E	G	H	I
1989	0.260	0.240	0.262	0.152	0.248	0.081	0.204	0.145
1999	0.334	0.233	0.240	0.205	0.171	0.119	0.243	0.371
2009	0.321	0.475	0.269	0.430	0.272	0.144	0.383	0.333
2019	0.306	0.485	0.305	0.251	0.331	0.214	0.309	0.286

## Data Availability

Not applicable.
